# Aspects of the Material Characteristics of the Filtration Systems of a Milling Plant in the Southeastern Area of Romania

**DOI:** 10.3390/foods14071202

**Published:** 2025-03-29

**Authors:** Liliana-Caliopi Iscru, Gheorghe Voicu, Elena-Madalina Stefan, Gabriel-Alexandru Constantin, Alina-Daiana Ionescu, Paula Tudor

**Affiliations:** Department of Biotechnical Systems, Faculty of Biotechnical Systems Engineering, National University of Science and Technology POLITEHNICA Bucharest, 060042 Bucharest, Romania; lyly_lylyk@yahoo.com (L.-C.I.); stefanelenamadalina@gmail.com (E.-M.S.); gabriel.constantin@upb.ro (G.-A.C.); daianaionescu_22@yahoo.com (A.-D.I.); paula.tudor@upb.ro (P.T.)

**Keywords:** wheat milling, bag filter, dust, physical properties, granulometric analysis

## Abstract

This paper presents the dedusting systems of a wheat mill in SC Agro Chirnogi, located in the southeastern area of Romania, and the physical characteristics of the material at the inlet, specifically at the base of the bagfilter systems. Samples were taken from both the dust removal system of the wheat cleaning technological phase (before milling) and from the dust removal system of the mill itself. It was found that the average particle size of the material at the cleaning section filter is between 1.191 and 0.563 mm, and the particle size of the material at the grinding section filter is between 0.278 and 0.186 mm, with their particle size distribution mainly following an exponential Rosin–Rammler distribution. Also, the bulk density of the material in the two bag filters ranges between 401 and 667 kg/m^3^ at the inlet and between 452 and 632 kg/m^3^ at the outlet of the cleaning technological phase filter. At the mill grinding section filter, the bulk density was between 426 and 506 kg/m^3^ at the inlet and between 422 and 580 kg/m^3^ at the outlet. The density of the material was between 854 and 1282 kg/m^3^ for the last fractions at the exit of the cleaning section filter and between 1309 and 1323 kg/m^3^ at the grinding section filter. Determinations were also made for other characteristics.

## 1. Introduction

In the grain milling process, pneumatic transport is widely used between the processing machines. In addition, most of the machines in the mill are equipped with dust extraction windows; however, in both cases, when the air is discharged into the atmosphere, it must be cleaned of small particles of suspended material (dust) [1].

This is also a safety measure because, under certain conditions and concentrations, the dust in cereal flour can become explosive. At the same time, the dust can cause health problems for operators in the mill’s technological flow.

To ensure air purification, a filtration system is installed at the end of the mill’s transport or suction system, consisting of an assembly of cyclones and bag filters. The separation between the solid and gaseous phases is influenced by the size or density of the material particles.

The specialized literature in the field of air filtration in grain mills mainly analyzes the health problems of machine operators inside the mill, as well as the modeling and simulation of air flow in the pneumatic transport system, specifically inside cyclones or suction filters.

Solari F. et al. [2] analyze the computational fluid dynamics of air–material flow inside the mill bag filter using a simulation model that was validated on an experimental pilot plant. These studies were carried out in order to obtain a predictive fluid dynamic model for the design of the filtration plant.

The air pressure at the filter inlet is measured before the dust-laden air enters the filter compartment, and the air pressure at the outlet is measured after the air has passed through the bags and been filtered. A sensor mounted on the air inlet pipe indicates the filter loading pressure and can indicate whether there are any problems with ventilation or airflow. Another sensor, mounted on the top of the filter on the exhaust pipe, shows how well the air is flowing through the filter and whether there are any blockages in the bags.

Pulse jet bag filters are typically operated between two preset pressure differential levels across the filter, a maximum level and a minimum level. The pressure difference (Δp = p1–p2) is the most important indicator, as it shows the degree of clogging of the bags. A significant increase in the Δp indicates that the bags are clogged and need to be cleaned. The optimal choice of the two pressure levels is made based on the maximum ratio between the time interval for a filtration cycle and the number of air jet pulses during the filter cleaning step.

Krammer et al. [3] found that for a given upper pressure difference, the filter exhibits an optimal lower pressure difference at a relatively low level. However, various imperfections have been identified in pulsed-jet filters and are most evident in the decrease in pressure as a function of time [4]. The authors propose a new filter model that addresses these observed imperfections.

A pressure difference of Δp = 50–150 mm H_2_O (50–150 kPa) indicates the existence of a clean filter, Δp = 150–250 mm H_2_O (150–250 kPa) indicates a half-loaded filter, and a Δp over 250 mm H_2_O (>250 kPa) indicates that the filter needs cleaning. If the Δp is too small, it may indicate air leaks or perforated bags, and if the Δp is too large, the bags are saturated and need to be cleaned or replaced.

In their paper, Saleem et al. [5] provide experimental data from a pilot-scale pulse jet regenerated bag filter test facility for three types of needle felt using air and limestone dust under ambient conditions. The experimental results presented in the paper show that the specific resistance of the filter media is independent of speed, while the specific resistance of the filter cake exhibits a linear variation with the speed of filtration.

Also, filtration curves vary depending on the initial pressure inside the filter and the nozzle diameter of the bag jet cleaning system [6]. The filtration time increases with increasing initial internal pressure or larger nozzle diameter. However, there is a critical value of the pressure inside the filter that is necessary for effective bag cleaning, as well as an effective critical value of the residual pressure loss for the pulse jet cleaning system.

The filter elements are encased in a metal or plastic cage to prevent movement during operation, with dust holding capacity being the most important parameter for filter design. Depending on the construction of the bag—regardless of the material it is made of or whether it is multilayer or pleated—the dirt holding capacity is significant, with a pleated bag having a larger filtration area [7].

For pleated filter elements, the ratio between the pleat height and pleat pitch has an influence on the filter cleaning mode (either at fixed time intervals or for preset pressure drops).

In the case of filtration, the regeneration of the filter elements is achieved by a countercurrent fluid flow through the porous medium of the bags, which removes the dust particles retained by the filter cloth [3,8].

The design of the cleaning nozzles and Venturi nozzles is particularly important for achieving the pressure impulse necessary for the efficiency of bag cleaning and the removal of the dust layer from the bags [8]. The same authors show the need for Venturi nozzles in low-pressure filters, whose role is to limit the reflux that could create problems during bag cleaning. They designed a 3D CFD model to simulate transient cleaning with an impulse jet, which was validated using experimental measurements at low pressure (2 bar).

The authors of paper [9] show that timely cleaning performs better for high-fold ratio filters, while on-demand cleaning is preferable for low-fold ratio filters. Also, internal pressure is critical for cleaning filter cartridges, but pulse duration has little effect on cleaning efficiency. However, pulse jet cleaning could influence particle penetration through the filter media.

The performance of a pulse jet baghouse filter can be evaluated, as previously stated, by the filtration speed and the filter pressure drop at the beginning of bag cleaning. In general, the effective residual pressure loss is determined to indicate the cleaning performance after the pulse jet is applied.

In paper [10], the authors found that there is a critical average pulse overpressure beyond which the cleaning performance of filter bags does not improve, and they determined that the final filtration resistance of the bags is the parameter for deciding whether Venturi nozzles are necessary for better cleaning performance. It was found that the use of Venturis is not recommended for a final filter resistance coefficient lower than 50 kPa·s·m^−1^.

To control and maintain the proper functionality of the bag filter system, a control system based on PLC or modular control boards is used. Debnath et al. [11] have designed a dust collector controller that can be installed by any user, with or without programming knowledge, featuring main control keys for pulse duration and pulse interval. The system operates solenoid valves that sequentially release air pressure on the filter bags; the pulse duration can be adjusted in the range of 0.01 to 0.9 s, and the pulse interval can be set from 1 to 99 s.

There are authors who propose the use of Digital Twin technology for monitoring and controlling industrial systems, a technology that involves 3D modeling through a virtual replica of these systems. Thus, Solar et al. [12] integrate real data from an air suction system with the dynamic simulation of one-dimensional fluids to develop a digital twin for its real-time control and regulation.

They found that it is necessary for the airflow to be correctly distributed among the different branches of the system, requiring frequent balancing, since the operating conditions of these systems are constantly changing, with the continuous accumulation of solid particles on the filter elements of the bag filter causing the pressure to decrease and the flow to change continuously. For this reason, they developed a digital model of the installation that enabled accurate and real-time calculation of the airflow drawn by the different branches of the system.

Various dust collection and exhaust systems have been proposed, with Xie et al. [13] proposing, among other things, a cyclone equipped with a filter cartridge before the air is discharged into the atmosphere. The results of the paper show that the cartridge filter cyclone has a separation efficiency of about 99.86% at a feed rate of 50 m^3^·h^−1^, which is about 15.32% higher than that of a conventional cyclone. The equipment proposed by the authors has, in particular, a separation efficiency for 2.5 μm particles of 99.11%, which is 70.80% higher than that of a conventional cyclone and 0.08% higher than that of a filter cartridge. In addition, the cleaning interval of the filter in the cartridge cyclone increases compared to that of a simple cartridge filter.

The grinding characteristics of wheat in industrial grinding plants are presented in detail in the paper [14], where the authors describe the technological phases of the grinding process, the components resulting from the process, and the method of analyzing them.

Wheat flour particles with an average size of 84 μm exhibit a maximum explosion pressure of 0.700 MPa at a concentration of 0.600 kg·m^−3^, with the explosion constant being 16.9 bar·m/s, while wheat flour particles with an average size of 50 μm exhibit a maximum explosion pressure of 0.797 MPa at a concentration of 1.000 kg·m^−3^, with the explosion constant being 5.49 MPa·m·s^−1^. The results presented show that the particle size distribution has a significant influence on the explosion distribution of flour [15].

The causes of explosions in industrial facilities (including flour milling) can be multiple, as shown in the paper [16].

In addition, the issue of suspended dust particles in industrial mills is analyzed from the point of view of the health of workers in the technological flow, especially concerning relatively small particles (PM10, PM5, PM2.5) across a wide range of papers [17,18,19,20]. The findings indicate that the cleaning technological phase is the most contaminated area of a mill.

In our paper, we present the dust removal systems of a wheat mill in the southeastern area of Romania and the physical characteristics of the material resulting from the system’s feeding and discharge, specifically what is recovered at the base of the dust removal system. Samples were taken from both the dust removal system of the wheat cleaning before grinding and the dust removal system of the mill itself. Among the characteristics analyzed, we analyzed the particle sizes and their granulometric distribution, as well as the bulk density and the density of the material and its components, along with the coefficient of angle of repose. By comparing the material from the feed and that from the base of the filter housing, conclusions can be drawn about the material discharged from the system into the atmosphere. The results of our experiments can be useful to specialists and operators in the grain processing technological flow in order to properly adjust the dedusting equipment and systems.

## 2. Materials and Methods

**Figure 1:**
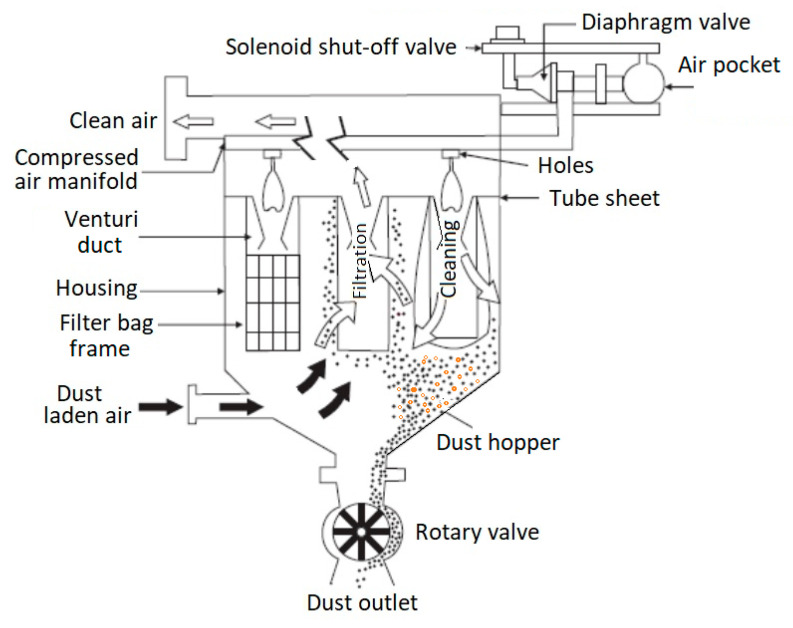
Pulse jet bag filter, (adapted from [19,21]).

Low-pressure bag filters operate according to the principle shown in Figure 1, with the air–dust mixture entering tangentially from the bottom. After the mixture is filtered through the filter bags, the air exits from the top, and the material that does not pass through the bag fabric falls into a collecting hopper, from which it is periodically evacuated through a sluice.

**Figure 2:**
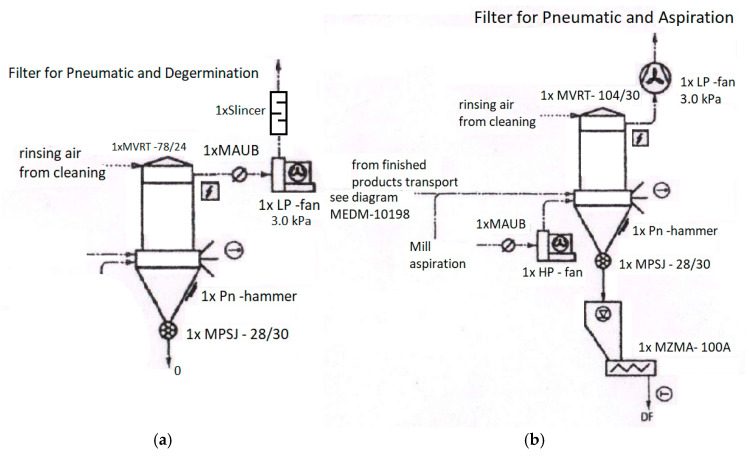
Dust collection systems before air discharge into the atmosphere: (**a**) for the grain cleaning sector; (**b**) for the actual grinding sector of the mill.

In the wheat cleaning sector before milling, the Agro Chirnogi mill in Romania is equipped with a MVRT 78/24 dust collector (Buhler filter) (Figure 2, left), manufactured by Buhler in Uzwill, Switzerland. This collector contains 78 filter bags, each measuring 120 mm in diameter and 2.4 m in length, with a total filter surface area of 44.7 square meters. The filter is equipped with a 3 kPa pressure exhaust fan and a 45 kW electric motor, with a flow rate of about 3 m^3^/s.

This filter is suitable for air volumes between 234 and 468 m^3^/min, depending on the specific filter load.

Cleaning is performed using an air pulse process that removes dust particles from the filter bags. As dust particles accumulate on the filter bags, air resistance increases. A pressure sensor monitors the pressure difference between clean and contaminated air. When this pressure difference reaches a certain threshold, an automatic pulse system triggers jets of compressed air through the cleaning tubes. The compressed air is suddenly introduced into the filter bags, which causes the dust particles to detach and fall. The detached particles then fall into the collection container located at the base of the filter, which is periodically emptied to prevent excessive dust accumulation, as shown in Figure 1 and Figure 2.

The bag filter collection system can also be equipped with a pnematically operated hammering mechanism to loosen the accumulated dust, which hits the collection hopper at regular intervals.

The filter bags of the MVRT filter are usually made of special, dust- and wear-resistant textile materials, such as PP/PE, with operating temperatures below 90 °C. The PP/PE filter bags are usually non-woven, offering an optimal porous structure, high durability, and a smooth surface for more efficient cleaning by compressed air pulses. At the outlet of the dust collector, there is an MPSJ-28/30 airlock.

The diagram in Figure 2, on the right, shows the main dust removal system of the mill, which is used for both the pneumatic transport of grist intermediate products and the dust suction from the processing equipment area. The filter house is equipped with 104 bags, each 120 mm in diameter and 3 m long.

The bag filter material is antistatically treated to prevent dust accumulation and improve air cleaning. It has a pore size of around 20–50 µm for the bag filter in the grinding section and 30–80 µm for the bag filter in the grinding preparation section.

On the schematics, the MAUB represents a butterfly valve used at the end of an air collection line, upstream of the high-pressure fan, to reduce the peak current of the fan motor at start-up. This allows the fan to quickly reach operating speed without having to overcome air resistance.

Next, the samples were processed and analyzed by particle size using an Analysette 3 Spartan Vertical Vibrating Laboratory Sieve Shaker (Fritsch, Idar-Oberstein, Germany) sieve classifier. After several attempts to choose the appropriate sieves (in terms of choosing the top sieve, so that between 3–10% of the sieved amount remained on it, and the next sieve had apertures about 1.41 times smaller), the sieves listed in Table 1 were chosen. This table also presents the percentages of material resulting from sieving a quantity of 0.100 kg of material, as previously specified, collected on each sieve over a duration of 3 min. Also, the cumulative amounts of material that passed through the sieve, as well as those that were rejected by the classifier sieves, are presented (see Table 1). Two determinations were made for each material category (inlet–outlet filter), but the data were retained for only one determination, after it was found that the fractions obtained were very close in appearance and mass. The dimensions of the sieve apertures used for sifting were between 2.0 and 0.315 mm for the material collected at the cleaning section filter, and between 0.630 and 0.100 mm for the material collected at the grinding section filter. The amplitude of the vibrations of the sieve classifier was about 3 mm because it was found that at lower values, the sieving was not complete.

The average particle size of the material mixture, regardless of the collection point, is calculated using the following relationship [22]:

(1) $$d_{m}=\frac{\sum_{i=0}^{n}p_{i}\cdot d_{i}}{\sum_{i=0}^{n}p_{i}},$$

where *p_i_* is the percentage of material on the sieve, *i*; *d_i_*—the average diameter of the particles of the fraction on the sieve, *i* (considered the arithmetic mean of the aperture sizes of the sieves that frame the fraction, *i*), and *Σp_i_* = 100 (because the sample mass was 0.100 kg).

**Figure 3:**
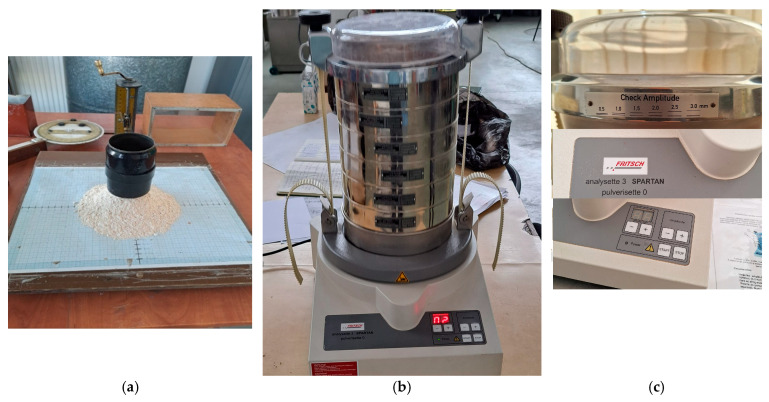
Laboratory stand for determining the coefficient of angle of repose for the material (**a**) and the sieve classifier: overview (**b**) and details (**c**).

Based on the data collected at the classifier sieves and presented in Table 1, curves were plotted for the cumulative percentages of material (T%) that passed through the apertures of the sieve above and the cumulative percentages of material that were rejected by the sieve below the current sieve (R%) (see Figure 3).

The graphic representation of these curves can provide us with information regarding the correct choice of sieves. The selection of these sieves may not always correspond to the specialized literature, even if an attempt is made to follow the guidelines (*d_i_ = √2·d_i−1_*, where *d_i_* is the mesh size of the sieve, *i*, and *d_i−1_* is the aperture size of the sieve below it), because the material collected at the inlet and outlet of the two filters is very inhomogeneous. Therefore, intermediate fractions with values that are very different from the others will be observed. For a more complete analysis, the number of sieves in the classifier would have to be very large to ensure that the workload remains the same, and the result is not necessarily conclusive.

In the sifting process, the undersized particles are those that pass through the apertures of a sieve, and the oversized particles are those that have dimensions larger than the aperture of a sieve. From the analysis of the curves for undersized particles (as well as for oversized particles) for the four material samples analyzed, it is found that they can generally be expressed using exponential functions of the type presented as follows [22]:

-    Exponential function:

(2) $$y=y_{o}+A\;e^{-x/t},$$

-    Chapman function:

(3) $$y=a{\;\left(1-e^{-b\;x}\right)}^{c},$$

-    Rosin–Rammler function:

(4) $$y=100\;e^{-b\;x^{n}},$$

In this regard, regression analysis of the undersize particles’ cumulative curves was performed in order to establish the degree of concordance between the experimentally obtained data and the functions proposed for the analysis. This can be performed by analyzing the values of the coefficient of determination, R^2^, as well as the independence coefficient, χ^2^.

In continuation of the analyses carried out by the authors, the volumetric mass of the material on the classifier sieves was determined using a graduated beaker. After each fraction had been properly homogenized, the material was weighed in the graduated beaker and related to the volume specified by the beaker. Each operation was repeated twice in order to avoid any inconsistencies. Also, for some fractions of material from the filter outlet of the cleaning section and for the entire material from the filter of the grinding section, the density was determined using a 25 mL pycnometer with a capillary tube. A Kern electronic balance with a precision of 10^−4^ kg, filter paper, and xylene with a density of 870 kg·m^−3^ were used as the working fluid.

Due to the large volume of work and xylene consumption, for the material collected from the filter of the grinding section of the Agro Chirnogi mill in Romania, only the overall density was determined (not by fractions). However, pertinent conclusions canbe drawn based on the two values obtained.

For the four fractions for which the density or the volume mass was determined from the cleaning section filter and the grinding section filter (inlet and outlet), the porosity of the material could also be determined using the following relationship:

(5) $$\varepsilon \left(\%\right)=\left(1-\frac{\rho_{v}}{\rho }\right)\cdot 100\;(\%),$$

where *ρ_v_* is the volumetric mass, and *ρ*—the density of the material, both of which are expressed in kg·m^−3^ [22].

Finally, we present the method for determining the coefficient of the angle of repose using the laboratory cylinder method, as can be seen in Figure 3. This involves measuring the radii of the material layout on the base plate covered with graph paper (in four perpendicular directions), as well as the layout height of the cylinder base after lifting, ensuring that the tip of the cone is at the same horizontal level as the base.

Using the average particle diameter, *d_m_*, the specific external surface area, *S_e.m_.*, can be calculated using the following relation [22]:

(6) $$S_{e.m.}=\frac{6}{\rho \;d_{m}}\;(m^{2}/\mathrm{kg})$$

where *ρ* is the particle density, which is determined using a pycnometer.

## 3. Results and Discussion

Following the diagrams in Figure 2, material samples were taken in October 2024 from the feeding of the filtration systems, as well as from their emptying. The moisture content of the material taken from the mill cleaning filter was 8.22% at the filter feeding and 11.22% at the filter outlet, on a wet basis. At the same time, the moisture content of the material taken from the grinding section filter was 11.53% and 12.63%, respectively, also on a wet basis (a Kern moisture analyzer was used). The moisture differences between the material from the filter feeding and the material from the filter outlet are not significant; they fall within the recommended range for milling processing, especially since before milling, the wheat is humidified in order to achieve the moisture content of 16–16.5% necessary for processing. We also consider the fact that the material that enters the filter enclosure at any given time is not the same as that collected immediately upon discharge.

**Figure 4:**
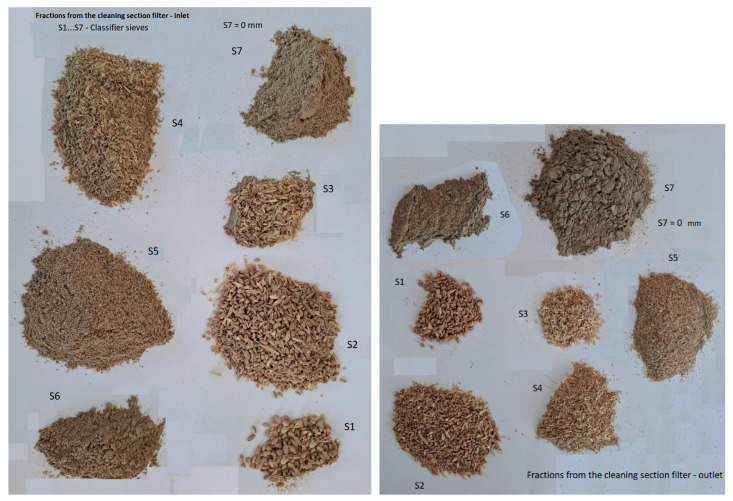
Fractions collected from the cleaning and degermination section filter: inlet and outlet (at different times).

**Figure 5:**
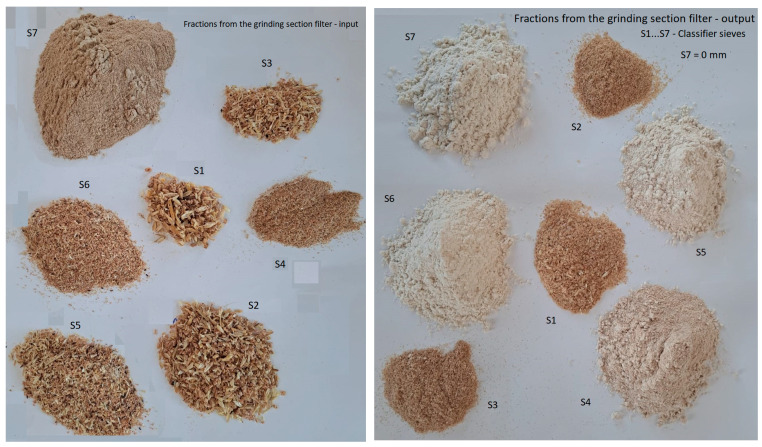
Fractions resulting from the granulometric analysis of the material collected from the grinding section filter: inlet and outlet (at different times).

The appearance of the fractions obtained from the granulometric analysis of the material processed at the two filters is presented in Figure 4 and Figure 5. It is important to note that the samples were not taken simultaneously, and the material that reaches the bag filter and is collected at the base of the filter is not evacuated immediately; therefore, the external appearance of the fractions is not necessarily the same.

It can be observed, however, that the material separated and collected on the classifier screens is quite coarse at the upper screens, including whole seeds or broken seeds, while at the lower screens, the material is much finer. The color of these fractions depends on the percentage of coating, mineral dust, and flour that reaches the filter at a given time. Thus, at the filter of the cleaning and degermination section, the last fractions have a much darker color, while at the bag filter of the grinding section, the fine fractions have a much lighter color, perhaps even a flour-like appearance, depending on the flour dust drawn in at a given time.

The results obtained from the granulometric analysis of the material collected at the four points (two from the cleaning section filter and two from the grinding section filter) are presented in Table 1.

The data in Table 1 show significant differences between the sizes of the material entering the filter and the sizes of the material collected at the bottom of the filter in the collecting hopper. This is mainly due to the fact that they were not collected strictly simultaneously and also due to the fact that the material subjected to filtration accumulates in the collecting hopper and is discharged only periodically, leading to the differences in the values in the table. In addition, the choice of a large number of screens was not possible, not only due to the large volume of work but also due to the high degree of dispersion in the particle sizes of the material.

After calculating the average particle size of the material mixtures at the inlet and outlet of the filter house, differences are also observed. Thus, using relation (1), average values between 0.563 and 1.191 mm were found for the material collected from the mill cleaning filter, and values between 0.186 and 0.287 mm were found for the material collected from the grinding section filter.

**Figure 6:**
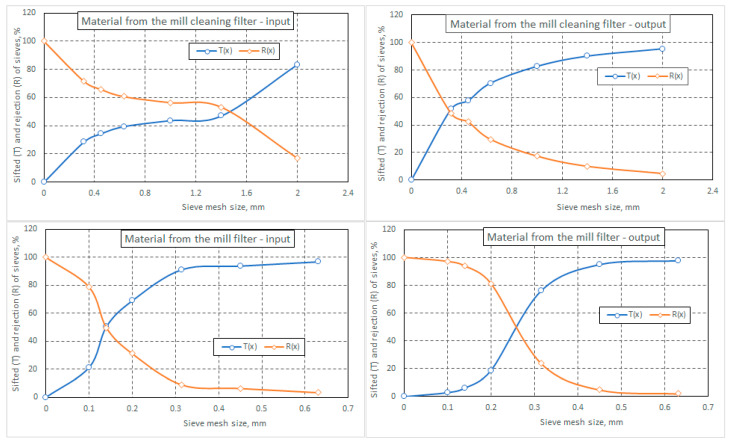
Cumulative percentages of material that passed through the sieve aperture—undersized particles (T%)—and material that was rejected by the sieves—oversized particles (R%).

The graphs in Figure 6, which were plotted based on the masses (percentages) of material collected on the six sieves of the classifier and in the box below them (S7), show the curves for the cumulative undersized particles (T%) and the oversized particles (R%). An increasing trend is observed in the cumulative undersized particle curve; however, this trend is not always continuous, as, for example, we have seen with the material at the bottom of the grinding section filter.

From the graphs, it is also observed that by vertically summing the values of T (%) and R (%), the total percentage of material results in 100% (corresponding to 100 g of sieved material).

Also, from the analysis of Table 1, it can be noted that there are some differences (not conclusive, as stated previously) between the amounts of material on similar sieves of the classifier for the material at the filter inlet and the filter outlet (see Table 2). However, we can state that the percentage of material with the smallest dimensions (collected at the base of the classifier) decreases, which would lead us to state that some of the particles in these fractions (being very fine) pass through the filter bag material. Therefore, we would recommend the inclusion of another system for the (final) collection of these dust particles from the clean air exhaust pipe into the atmosphere.

**Figure 7:**
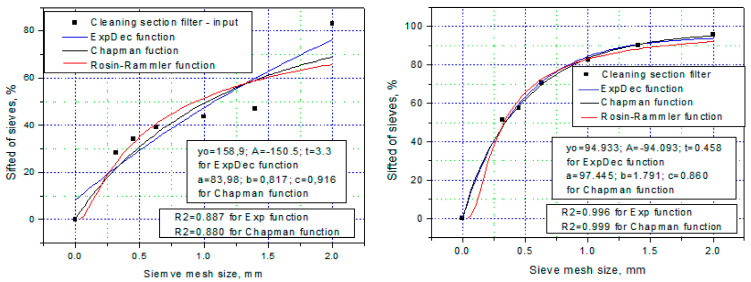

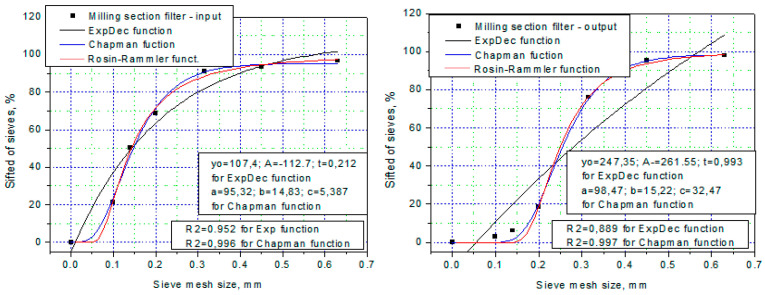
Cumulative distribution curves calculated using relations (2–4) and experimental points for the material collected at the filters of the cleaning/degermination section (input-output) and the grinding section (input-output) of the Agro Chirnogi mill.

Next, after performing a regression analysis in the Microcall Origin 8.0 program (OriginLab Corporation, USA), the graphs resulting from this analysis are shown in Figure 7. The coefficients of the regression functions, together with the values of the coefficient of determination R^2^ and the respective χ^2^, are presented in Table 3.

Analyzing the data in Table 3, as well as the regression curves in Figure 7, it is observed that not all functions (relations (2–4)) fit the experimental data equally well, which is mainly due to the values of the coefficients R^2^ and χ^2^. It is worth noting, however, that the high values of the coefficient of determination, R^2^, in most cases above 0.95, indicate that over 97% of the total variation in the percentage of material collected cumulatively on the sieve is correlated with the independent variable, i.e., the size of the sieve aperture. At the same time, it can be seen that many samples (regression curves) exhibit a coefficient χ^2^ with a low value, which allows us to conclude that those functions (which yield these values) correlate the experimental data well enough. This is very easy to see in the graphs.

**Figure 8:**
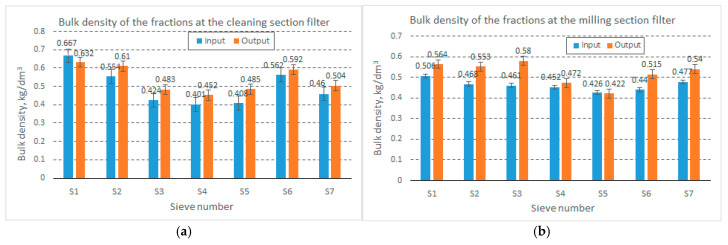
Bulk density of material collected from the cleaning section (**a**) and milling section (**b**).

**Figure 9:**
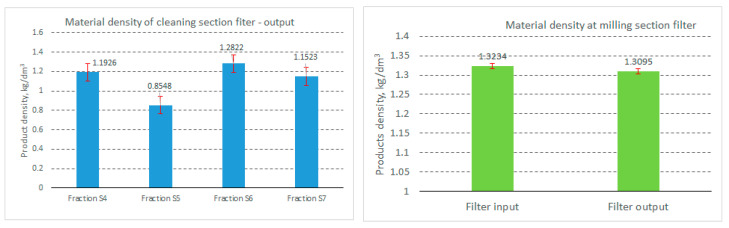
Density of material for four fractions collected from the outlet of the cleaning filteras well as for the material from the inlet and outlet of the filter collected from the grinding section.

As presented in the previous section, the results obtained from determining the bulk density of the material processed on the classifier screens are presented in Figure 8, and the density of the material is shown in Figure 9.

As can be seen in Figure 8, the volumetric mass of the material collected at the two filters of the entire wheat mill appears to have relatively high values but is much lower compared to the volumetric mass of the seeds or flour resulting from the milling process. Thus, in the wheat cleaning (and degermination) section, the volumetric mass of the material fractions fell within the range of 401–667 kg/m^3^ at the filter inlet and 452–632 kg/m^3^ at the filter outlet, while in the large filter (the grinding section filter), the volumetric mass of the material fell within the range of 426–506 kg/m^3^ at the filter inlet and 422–580 kg/m^3^ at the filter outlet (in both cases, however, the measurements were taken at different times; we specify that the times did not differ by more than 60 min).

Based on the percentages of material collected from the classifier screens, it can be estimated that the material processed by the cleaning section filter has a density of 555 kg/m^3^ at the filter inlet and 570 kg/m^3^ at the filter outlet, and the material processed by the grinding section filter has a density of 470 kg/m^3^ at the filter inlet and 484 kg/m^3^ at the filter outlet.

As shown in Figure 9, the material density of the fractions resulting from the granulometric analysis of the material collected at the base of the cleaning section filter (Figure 9, left) ranged between 854 kg/m^3^ for the fraction resulting from sieve S5 and 1282 kg/m^3^ for sieve S6 of the sieve classifier (sieves S4–S7 are located at the base of the sieve assembly, with the mesh sizes in descending order, and sieve S7 is, in fact, the bottom collecting box). This allows us to state that these fractions mainly contain fine flour particles, as well as fine particles of mineral dust drawn from the seed cleaning areas.

As can be seen from Figure 9 (right), as well as from the image in Figure 4, the processed material contains a significant amount of flour dust, and the values obtained are close to the density of flour, namely, 1323 kg/m^3^ at the filter inlet and 1309 kg/m^3^ at the filter outlet (obviously at different times).

Therefore, for the fractions obtained at the base of the classifier (S4–S7) from the outlet of the cleaning section filter (as shown in Figure 9, on the left), the porosity of the material, calculated using relation (5), ranged between 41.3 and 57.3%. In contrast, for the material collected at the grinding section filter, the porosity of the entire material was 64.5% at the filter inlet and 63.0% at the base of the bag filter.

Determined in the laboratory using the cylinder method, the coefficient of the angle of repose for the material collected at the filter of the mill cleaning section was approximately 43.49 degrees at the filter entrance and 41.71 degrees at the base of the filter (which, as can be seen, are very close values). In contrast, for the material collected at the filter of the mill grinding section (large filter), the coefficient of the angle of repose was 44.62 degrees at the filter entrance and 38.26 degrees at the filter exit, after two rows of samples.

By using relation (6), the value of the external surface area of the particles, *S_e.m_*_._, from the grinding section filter was determined to be about 24.37 m^2^/kg for the material sample collected at the filter feed and 16.48 m^2^/kg for the material sample collected at the filter outlet. For the S4–S7 fractions of the material collected at the filter outlet of the cleaning section, taking into account the average diameters of the classifier sieve openings from which they were obtained, the values of the external surface area of the particles were 6.17, 12.99, 12.23, and 40.34 m^2^/kg, which are relatively high values because the particles were obtained on the sieves with the smallest apertures.

Correlating operational parameters, such as suction speed/flow rate, the time periods at which the filter bags are unclogged, and the method of discharging the material from the base of the filters, with the properties of the material that feeds the bag filters can lead to the optimization of the performance of the filters and the process itself.

## 4. Conclusions

According to the results presented, we can draw the following conclusions:

(1) Material samples were taken from the filter inlet and outlet, and the samples were analyzed from a granulometric point of view. The granulometric curves for the undersized and oversized particles of the classifier sieves were drawn. The average particle sizes of the material collected at the cleaning section filter ranged between 1.191 and 0.563 mm, and the average sizes of the material particles processed at the grinding section filter ranged between 0.278 and 0.186 mm (inlet–outlet).
(2) The material collected at the inlet and outlet of the filters in the basic sections of the mill (cleaning, grinding) mainly follows an exponential distribution of the Rosin–Rammler or Chapman type, with values of the coefficients of determination, R^2^, mainly above 0.95 and independence, χ^2^, values under 9.5 (for both the Rosin–Rammler and Chapman functions).
(3) The bulk density of the material in the two bag filters ranges between 401 and 667 kg/m^3^ at the inlet and between 452 and 632 kg/m^3^ at the outlet of the cleaning technological phase filter. At the mill grinding section filter, the bulk density was between 426 and 506 kg/m^3^ at the inlet and between 422 and 580 kg/m^3^ at the outlet. Also, the density of the material was between 854 and 1282 kg/m^3^ for the last fractions at the exit of the cleaning section filter and between 1309 and 1323 kg/m^3^ at the grinding section filter.
(4) Maintaining the proportions of the material collected on the six sieves of the classifier, it can be estimated that PM10 could have values between 4.53 and 6.22% at the inlet of the grinding section filter and between 0.084 and 0.096% at the outlet of the same filter. For the cleaning section, the values are higher; however, according to the appearance of the fraction collected at the bottom of the classifier and according to our estimates, between 8.15 and 26.52% of the particles have sizes below 30–40 μm.
(5) Determinations were made for the coefficient of angle of repose of the material collected at the feed and discharge of the filters, as well as for the porosity and external surface of the material. Knowledge of these factors, as well as those mentioned above, is particularly important, mainly for adapting the technological process of wheat processing on the technological flow to the characteristics of the raw material and for adjusting the parameters of the installations in the flow.
(6) The authors recommend adding a final dust collection system with sizes smaller than the pore size of the bag fabric immediately after each of the two filters, before exhausting the air into the atmosphere, to collect PM10–PM2.5 material particles.

## Figures and Tables

**Table 1:** Results of particle size analysis.

Cleaning Section							Milling Section						
d_sieve_ mm	Input			Output			d_sieve_ mm	Input			Output		
	p%	T%	R%	p%	T%	R%		p%	T%	R%	p%	T%	R%
0	51.5	0	100	28.4	0	100	0	21.3	0	100	2.9	0	100
0.315	6.1	51.5	48.5	5.8	28.4	71.6	0.100	29.2	21.3	78.7	3.3	2.9	97.1
0.450	12.9	57.6	42.4	5.1	34.2	65.8	0.140	18.4	50.5	49.5	12.5	6.2	93.8
0.630	12.2	70.5	29.5	4.3	39.3	60.7	0.200	22.2	68.9	31.1	57.3	18.7	81.3
1.000	7.4	82.7	17.3	3.3	43.6	56.4	0.315	2.5	91.1	8.9	19.1	76	24
1.400	5.3	90.1	9.9	36.3	46.9	53.1	0.450	3	93.6	6.4	2.8	95.1	4.9
2.000	4.6	95.4	4.6	16.8	83.2	16.8	0.630	3.4	96.6	3.4	2.1	97.9	2.1
d_m_, mm	1.191			0.563			d_m_, mm	0.186			0.278		

p (%)—the percentage of material remaining on each sieve of the classifier; T (%)—cumulative percentage of undersized particles; R (%)—cumulative percentage of oversized particles, (%).

**Table 2:** Differences between the material entering the filter and that collected at the filter exit.

Mill Cleaning Section				Milling Grinding Section			
d_sieve_, mm	Input, %	Output, %	Difference, %	d_sieve_, mm	Input, %	Output, %	Difference, %
0	51.5	28.4	23.1	0	21.3	2.9	18.4
0.315	6.1	5.8	0.3	0.100	29.2	3.3	25.9
0.450	12.9	5.1	7.8	0.140	18.4	12.5	5.9
0.630	12.2	4.3	7.9	0.200	22.2	57.3	−35.1
1.000	7.4	3.3	4.1	0.315	2.5	19.1	−16.6
1.400	5.3	36.3	−31	0.450	3	2.8	0.2
2.000	4.6	16.8	−12.2	0.630	3.4	2.1	1.3

**Table 3:** Values of the coefficient of the regression relationships and the coefficient of determination, R^2^.

No.	Regression Function	Collecting Point	y_o_	A	t	R^2^	χ^2^
1	Exponential function $y=y_{o}+A\;e^{-x/t}$	Cleaning input	158.9	−150.5	3.3	0.887	104.22
2		Cleaning output	94.933	−94.093	0.458	0.996	6.53
3		Milling input	107.4	−112.7	0.212	0.952	104.48
4		Milling output	247.3	−261.5	0.993	0.889	339.21
		**Collecting point**	**a**	**b**	**c**	**R^2^**	**χ^2^**
5	Chapman function $y=a{\;\left(1-e^{-b\;x}\right)}^{c}$	Cleaning input	83.98	0.817	0.918	0.880	117.58
6		Cleaning output	97.445	1.791	0.860	0.999	5.08
7		Milling input	95.32	14.83	5.387	0.996	8.70
8		Milling output	98.47	15.22	32.47	0.997	8.03
		**Collecting point**	**b**	**n**	**-**	**R^2^**	**χ^2^**
9	Rosin–Rammler function $y=100\;e^{-b\;x^{n}}$	Cleaning input	0.662	−0.665		0.856	106.50
10		Cleaning output	0.187	−1.183		0.992	9.61
11		Milling input	0.0099	−2.185		0.998	4.26
12		Milling output	0.0026	−4.013		0.996	9.57

## Data Availability

The original contributions presented in the study are included in the article, further inquiries can be directed to the corresponding author.

## References

[1] Pranav P.K., Biswas M. (2016). Mechanical intervention for reducing dust concentration in traditional rice mills. Ind. Health.

[2] Solari F., Tagliavini G., Montanari R., Bottani E., Malagoli N., Armenzoni M. CFD model validation of a bag filter for air filtration in a milling plant. Proceedings of the International Food Operations and Processing Simulation Workshop 2017.

[3] Krammer G., Kavouras A., Anzel A. (2003). Optimization of pulse cleaning frequency during bag filter operation. Chem. Eng. Technol..

[4] Kavouras A., Krammer G. (2003). Distributions of age, thickness and gas velocity in the cake of jet pulsed filters—Application and validation of a generations filter model. Chem. Eng. Sci..

[5] Saleem M., Krammer G., Tahir M.S. (2012). The effect of operating conditions on resistance parameters of filter media and limestone dust cake for uniformly loaded needle felts in a pilot scale test facility at ambient conditions. Powder Technol..

[6] Lu H.-C., Tsai C.-J. (1998). A Pilot-Scale Study of the Design and Operation Parameters of a Pulse-Jet Baghouse. Aerosol Sci. Technol..

[7] Perlmutter B.A. (2015). Bag filter, Chapter 3—Types of Filtration Systems. Solid-Liquid Filtration.

[8] Andersen B.O., Nielsen N.F., Walther J.H. (2016). Numerical and experi-mental study of pulse-jet cleaning in fabric filters. Powder Technol..

[9] Lo L.-M., Chen D.-R., Pui D.Y.H. (2010). Experimental study of pleated fabric cartridges in a pulse-jet cleaned dust collector. Powder Technol..

[10] Tsai C.-J., Tsai M.-L., Lu H.-C. (2000). Effect of filtration velocity and filtration pressure drop on the bag-cleaning performance of a pulse-jet baghouse. Sep. Sci. Technol..

[11] Debnath T., Nazir I., Podder P.K., Sarker S. (2023). Design and implementation of a novel dust collector controller for powder production industry. World J. Adv. Res. Rev..

[12] Solari F., Lysova N., Montanari R. (2024). Monitoring and control of air filtration systems: Digital twin based on 1D computational fluid dynamics simulation and experimental data. Comput. Ind. Eng..

[13] Xie B., Li S., Jin H., Hu S., Wang F., Zhou F. (2018). Analysis of the performance of a novel dust collector combining cyclone separator and cartridge filter. Powder Technol..

[14] Voicu G., Biris S.S., Stefan E.M., Constantin G.A., Ungureanu N. (2013). Grinding characteristics of wheat in industrial mills, Chapter 15. Food Industry Book.

[15] Kuracina R., Szabova Z., Buranska E. (2019). Study of explosion characteristics of the wheat flour dust clouds in dependence of the particle size distribution. Res. Pap. Fac. Mater. Sci. Technol. Slovak Univ. Technol..

[16] Freschi F., Mitolo M., Tommasini R. (2017). Analysis of causation of a flour dust explosion in industrial plant. IEEE Trans. Ind. Appl..

[17] Sobczak P., Mazur J., Zawiślak K., Panasiewicz M., Żukiewicz-Sobczak W., Królczyk J.B., Lechowski J. (2019). Evaluation of dust concentration during grinding grain in sustainable agriculture. Sustainability.

[18] Stobnicka-Kupiec A., Górny R.L. (2017). Flour dust—Aspiration fraction documentation of proposed values of occupational exposure limits (OELs). Podstawy Metod. Oceny Sr. Pr..

[19] Dacarro A., Grisoli P., Del Frate G., Villani S., Grignani E., Cottica D. (2005). Micro-organisms and dust exposure in an Italian grain mill. J. Appl. Microbiol..

[20] Mishra J., Verma K., Mohanty S.K., Rath I. (2024). Health risks and dust exposure among flour mill workers in Eastern India: A comprehensive study. Curr. World Environ..

[21] Shah K.P. (2017). Working, Design Considerations and Maintenance of Bag Type Fabric Filters. https://practicalmaintenance.net.

[22] Constantin G.A., Voicu G., Stefan E.M., Munteanu M.G., Musuroi G., Voicea I. (2021). Dimensional characteristics of grist intermediate products obtained in the first two technological passages in the reduction phase of an industrial milling plant. E3S Web Conf..

